# Studying Health Outcomes in Farmworker Populations Exposed to Pesticides

**DOI:** 10.1289/ehp.8526

**Published:** 2006-02-16

**Authors:** Linda A. McCauley, W. Kent Anger, Matthew Keifer, Rick Langley, Mark G. Robson, Diane Rohlman

**Affiliations:** 1 University of Pennsylvania School of Nursing, Philadelphia, Pennsylvania, USA; 2 Oregon Health and Science University, Portland, Oregon, USA; 3 University of Washington, Seattle, Washington, USA; 4 North Carolina Department of Health and Human Services, Raleigh, North Carolina, USA; 5 University of Medicine and Dentistry of New Jersey, Newark, New Jersey, USA

**Keywords:** biomarkers, cancer, epidemiology, health outcomes, immigrants, neurobehavioral, neuropathy, pesticides

## Abstract

A major goal of studying farmworkers is to better understand how their work environment, including exposure to pesticides, affects their health. Although a number of health conditions have been associated with pesticide exposure, clear linkages have yet to be made between exposure and health effects except in cases of acute pesticide exposure. In this article, we review the most common health end points that have been studied and describe the epidemiologic challenges encountered in studying these health effects of pesticides among farmworkers, including the difficulties in accessing the population and challenges associated with obtaining health end point data. The assessment of neurobehavioral health effects serves as one of the most common and best examples of an approach used to study health outcomes in farmworkers and other populations exposed to pesticides. We review the current limitations in neurobehavioral assessment and strategies to improve these analytical methods. Emerging techniques to improve our assessment of health effects associated with pesticide exposure are reviewed. These techniques, which in most cases have not been applied to farmworker populations, hold promise in our ability to study and understand the relationship between pesticide exposure and a variety of health effects in this population.

The major goal of studying farmworkers is to better understand how their work environment, including exposure to pesticides, affects their health. Our understanding of the health effects associated with pesticide exposures is formed by contributions from toxicology, physiology, pharmacology, epidemiology, sociological studies, and the emerging area of “omics.” The purpose of this article is to examine the issues related to studying health effects associated with chronic low-dose exposure to pesticides particularly in the farmworker population. We present a brief overview of the range of health outcomes that have been associated with pesticide exposure. Then the basic tools of epidemiology and surveillance are discussed in the context of the farmworker population. The limitations and information gaps for conducting this research are described. We present neurobehavioral health effects as one of the best examples of an approach used to study health outcomes in farmworkers and the methodologic challenges of conducting these assessments in field investigations. We conclude with a discussion of emerging techniques that have the potential to improve our ability to study and understand the relationship between pesticide exposure and a variety of health effects in this population.

## Health Effects Associated with Pesticide Exposure

Organophosphate pesticides have gained popularity worldwide in preference to organochlorines, which are persistent and more damaging to the environment (Jaga and Dharmani 2003). Organophosphates are associated with well-known acute health problems such as nausea, dizziness, vomiting, headaches, abdominal pain, and skin and eye problems (Ecobichon 1996). Some studies have also indicated that pesticide exposure is associated with chronic health problems or health symptoms such as respiratory problems, memory disorders, dermatologic conditions, cancer, depression, neurologic deficits, miscarriages, and birth defects (Arcury et al. 2003; Cordes and Rea 1988; Daniels et al. 1997; Das et al. 2001; Engel et al. 2000; Eskenazi et al. 1999; Firestone et al. 2005; Garcia et al. 2003; Moses 1989; O’Malley 1997; Schwartz et al. 1986; Stallones and Beseler 2002; Strong et al. 2004; Van Maele-Fabry 2003). Daniels et al. (1997) provided a comprehensive review of the epidemiologic studies of links between pesticide exposure and cancer in children, but these studies were not with farmworker children, who may experience disproportionate risk of exposure and who may be very under-represented in cancer registries. Recent reviews by Alavanja et al. (2004), Kamel and Hoppin (2004), and Priyadarsi et al. (2000) have examined the link between pesticide exposure and neurologic outcomes and cancer, arguably the two major end points examined in organophosphate-exposed workers. In these extensive reviews, the authors point out that carcinogenicity and neurotoxicity reflect different mechanisms of toxicity that require different epidemiologic investigations to assess the effects.

The review by Alavanja et al. (2004) summarized studies examining the link between pesticide exposure and cancer. Non-Hodgkin lymphoma (NHL) has been one of the most extensively studied cancers, with more than 30 studies in the scientific literature. Associations between NHL and exposures to phenoxyacetic acid, organochlorine, and organophosphate compounds have been reported. Leukemia has also been studied extensively, again with more than 30 studies showing associations with insecticide and herbicide use. Similar associations have been shown with prostate cancer, multiple myeloma, and soft tissues sarcomas. There is less supportive literature of an association between pesticides and other types of cancer, although there is some literature of an association between chlorinated compounds and breast and testicular cancer and Hodgkin disease.

In the review by Kamel and Hoppin (2004) of the health effects of pesticide exposure, the authors report that chronic pesticide exposure is associated with a broad range of nonspecific symptoms, including headache, dizziness, fatigue, weakness, nausea, chest tightness, difficulty in breathing, insomnia, confusion, and difficulty concentrating. Many of the studies indicate that pesticide exposure is associated with deficits in cognitive function. There is also extensive literature supporting the association of Parkinson’s disease and other neurologic diseases and pesticide exposure. Kamel and Hoppin (2004) point out studies to date have been unable to identify specific associations between pesticide exposure and Parkinson disease risk.

Occupational exposure to pesticides and adverse reproductive effects have also been reviewed (Hanke and Jurewicz 2004). Many pesticides known to have reproductive effects are no longer used in the United States, but employment in agriculture appears to be associated with specific morphologic abnormalities in sperm, and studies suggest that parental employment in agriculture could increase the risk of congenital malformations in offspring, particularly orofacial cleft, as well as musculoskeletal and nervous system defects. The authors also report that studies are unequivocal on a relationship between occupational exposure to pesticides and infertility.

## Epidemiologic Challenges in Studying Health Outcomes in Farmworker Populations

To develop effective studies of long-term health outcomes in farmworkers and their children, tools and techniques used for epidemiologic studies must be made to function effectively. The basic components that are necessary to effectively study the association between pesticide exposure and health effects are determination of the population at risk; a valid determination of exposure; verification of diagnosis, symptom, or biological marker of a health effect among the populations being studied; methods to link individual exposure to health effects; and the ability to establish a temporal relationship between the exposure and the health effect. In attempts to study farmworker populations, these tools are often incomplete, dysfunctional, or nonexistent.

The number of total migrant and seasonal farmworkers (denominator data) is not well characterized (Villarejo 2003). Various methods have been employed to estimate the size of the farmworker population, and estimates range from 2.5 to 5 million (Hansen 2003; National Center for Farmworker Health 2004).

The difficulty of determining rates of pesticide illness is exemplified by the lack of ability to estimate the number of cases of acute pesticide illness. Although 30 states require reporting of occupational pesticide-related illnesses, many cases are not reported (Calvert et al. 2003). Only 8 states have surveillance programs for these illnesses, and poison control center data can also lead to underascertainment. At this time only 5 states have legislation requiring extensive reporting of pesticide use, and 4 of these states require growers to report pesticide use on crops. Data collected from these pesticide use reporting programs include product name, amount applied, location, and crop type. Pesticide use reporting systems can then be linked to episodes of pesticide illness, but clinicians often are not aware when pesticide illness reporting is required in their state (Connan 1996).

Data sources on the health effects of pesticides such as worker compensation (WC) systems and health insurance information systems are generally inaccurate for farmworkers. State WC systems, although required by federal law to exist, differ from state to state, and agriculture as an industry that is exempt in many states. Even in states where agriculture is not exempt, community clinicians may be discouraged from filing WC claims because of the time required for completion of paperwork and filing. A farmworker, who may not understand the WC system or his or her rights in it, may not be in a position to protest when claims are closed or are not filed.

Health insurance information, a potentially rich source of information for epidemiologic studies, functions poorly in this regard because most farmworkers lack health insurance. A study by the California Institute for Rural Studies indicated that, based on a health status study of 971 farmworkers and their families, nearly 70% of subjects lacked health insurance of any kind (Villarejo et al. 2000). The same study found that few workers received routine medical or dental care with or without insurance coverage.

An additional problem that limits the ability to quantify health issues in farmworkers is that Mexican farmworkers may return to Mexico to receive health care. One study found that families living along the border with Mexico received half of their health care in Mexico. This behavior took place regardless of insurance coverage (Seid et al. 2003). Many Mexican migrants will spend portions of the off season in Mexico and pursue health care for chronic problems at that time, denying U.S. data systems information on these conditions.

For farmworkers to be counted in the systems mentioned above as having pesticide-related illness, clinicians must both diagnose and report these illnesses. Most clinicians receive little training in occupational and environmental health (Graber et al. 1995; Schenk 1996). The National Strategies for Health Care Providers, a working group organized by the U.S. Environmental Protection Agency, concluded that clinicians do not generally receive specific training in diagnosing pesticide poisonings or other pesticide-related health effects (National Environmental Education and Training Foundation 2002). One study of Washington State clinicians demonstrated that few appeared to be well versed in the diagnosis or treatment of pesticide poisonings. Even clinicians from agricultural areas on average could identify only 75% of pesticide symptom questions correctly (Connan 1996).

The transient nature of farmwork may have important implications with respect to studies done using death certificates. Ample literature exists demonstrating that the job title listed on death certificates may inaccurately portray the work experience of the decedent (Olsen et al. 1990; Steenland and Beaumont 1984). The information on the certificate may be the most recent or most prestigious job rather than the principal job. Although a percentage of farmworkers may spend their entire lives in farm work, many will move from migrant or seasonal farm work to higher-paying, less physically demanding, and less mobile jobs as soon as possible. As a result, death certificates may not reflect the contribution of farm work to a worker’s total work life.

### Cohort and case–control studies

The ability to characterize long-term health outcomes, exposure variables, and confounders and the ability to follow subjects over time present specific challenges in studies of chronic health effects in farmworkers. Zahm and Blair (1993), in discussing the feasibility of cancer studies in this population, touched on many of the obstacles facing epidemiologic research in this realm. Subsequent work by these and other investigators explored several interesting approaches to several of these obstacles.

The mobility of the migrant and seasonal farmworkers presents a significant challenge to investigations of long-term health effects (Quandt et al. 2002). Establishing a true cohort has been overcome by some through the use of fixed housing opportunities such as the Northern California Migrant Family Housing Centers (McCurdy et al. 2002). Such fortuitous situations represent the exception rather than the rule for migrant farmworkers. The difficulty of such studies in these populations is rarely described in the literature (Quandt et al. 2002 is an exception). Follow-up studies that fail to achieve successful follow-up either appear in publication as cross-sectional studies or fail to appear at all. Publication bias toward successful studies tends to weed out the reports that would illustrate the difficulty of cohort follow-up in any population. Information on the difficulty of following migrant farmworker populations no doubt suffers the same fate. However, a pair of methodologic articles provides limited insight into the challenge of conducting long-term follow-up in farmworkers. In a study of Wisconsin workers, Nordstrom et al. (2001) attempted to locate 100 randomly selected farmworkers 10 years after their registration in a Wisconsin clinic. Only 6 of 100 could be located in Wisconsin, and the vital status could be ascertained on only 56% of 46 subjects after a moderately intensive search in the registered home-base state of subjects. Cooper et al. (2001) had remarkable success in relocating farmers enrolled in chronic disease clinic studies in Starr County, Texas, with 90.8% of subjects relocated 10 years after enrollment. These two follow-up studies may demonstrate the difference in success because of the start point for the follow-up search but, as Zahm and Blair (2001) point out, are probably a reflection of a variety of factors, including permanent residence, the quality of community contact, and the presence of a long-standing community-based research program.

There are currently several studies of farmworker populations that are attempting to study exposures during sensitive periods of childhood development and health outcomes in this population. Researchers from the University of California have established a birth cohort and are following the children longitudinally. Although the farmworker population they are targeting is not as mobile as all farmworkers, they have described the extensive methodologic issues in accessing and maintaining the cohort, including the need for culturally appropriate assessment tools and the use of community workers in recruitment and maintenance of the study sample (Eskenazi et al. 2005).

### Workplace considerations

In the conduct of studies on factors related to occupational exposures, such as pesticide studies, the work-place is often the best and most important venue for data collection. However, studies of farmworker populations rarely use the work-site as the source for study recruitment (Arcury et al. 2001; Eskenazi et al. 1999; McCauley et al. 2001; Thompson et al. 2003). The migratory nature of farmworker often precludes the possibility of conducting traditional occupational health studies in agricultural settings. The power differential between employer and the nonunionized farmworker (most farmworkers) plays an important role in determining the feasibility of worksite studies. Workers will rarely volunteer for a study if they perceive that such participation threatens their jobs.

### Language

Language preference among affected populations can be a substantial barrier to efficiently conducting population epidemiologic research on pesticide health effects. In the United States, the large majority of farmworkers are Spanish speaking, but increasing numbers of workers do not speak Spanish as their primary language. Farmworkers of other nationalities are also being seen McCauley et al. 2002; McDonald 2001). The National Center for Farmworker Health (2000) estimates that 84% of the migrant and seasonal farmworker populations speak Spanish as their primary language. A greater challenge is the presence of predominantly indigenous Mexican language speakers in the migrant workforce. Villarejo (2003) found that 8% of California farmworkers studied reported being of indigenous origin, and a small percentage spoke only indigenous languages. McCauley et al. (2002) found that a surprising 36% of adolescent farmworker subjects in Oregon spoke indigenous Mexican languages. Of particular importance is that several of these languages do not have a written form. Even when workers speak Spanish, low literacy limits a worker’s ability to respond to written material and interferes with participation.

Thus, multiple factors, some unique to the population and the work and others generalizable to deficiencies in our national surveillance systems, make study of health effects of pesticides, long and short term, difficult in farmworker populations. Hence, investigators have designed studies addressing these difficulties and have identified health effects in this population. Studies of neurobehavioral health effects are excellent examples of strategies that can be taken to conduct these studies.

## Neurobehavioral Health Effects

Neurobehavioral performance batteries are a well-recognized method of assessing potential health effects associated with pesticide exposure (Table 1), but the validity of the results obtained from the testing is dependent upon several laboratory quality-control concerns. These laboratory quality-control concerns are similar to and as crucial as the quality-control issues in measuring exposure variables. Use of these batteries with non-English–speaking immigrant populations presents additional challenges and could ultimately affect the interpretation of the study results.

### Laboratory capacity

Assessment of neurobehavioral performance requires specific laboratory support and resources. Specific training is needed so that the tests are administered in a standardized way to each participant. The World Health Organization Neurobehavioral Core Test Battery (NCTB) (Johnson et al. 1987) and the Adult Environmental Neurobehavioral Test Battery (Anger et al. 1994) rely on individually administered tests and require extensive training of the examiner to ensure standard administration across participants. Computerized tests batteries such as the Neurobehavioral Evaluation System (Letz 1990), the Behavioral Assessment and Research System (BARS) (Anger et al. 1996), and the Swedish Performance Evaluation System (Iregren et al. 1996) also require examiner training.

During the actual data collection period, quality-control checks are necessary to determine if the examiners follow the correct protocol as trained. A checklist that examines whether the correct protocol is being followed, forms are filled out correctly, and appropriate interactions are occurring between the testers and the study participants should be developed for each protocol and administered periodically throughout the study.

The resources available to implement neurobehavioral testing can also affect the number of participants that can be assessed in a study, a crucial factor of studies of farmworkers. Noncomputerized or paper-and-pencil tests can be administered only one-on-one to study participants. Furthermore, at the end of the study these data need to be manually entered into a database. Computerized testing allows the possibility of increasing the participant to examiner ratio. Ten adults with at least some high school education can be tested with only one examiner with the computerized BARS (Anger 2003; Rohlman et al. 2003). However, when testing farmworkers with < 6 years of education, it is possible to test only up to four participants with one examiner. With young children, a one-to-one ratio is necessary to help maintain motivation throughout data collection (Rohlman et al. 2001a). As children become older, their reading ability improves and they have more school and computer experience, so this ratio may be increased.

### Integrating neurobehavioral testing in the design of farmworker studies

Neurobehavioral outcome protocols typically study people at one point in time in a cross-sectional design, comparing the performance of exposed populations with either the performance of a control group or established normative data (e.g., Anger 1990; Anger et al. 1999). Finding a comparable control group is essential for an accurate interpretation of these results (e.g., Blair et al. 1996; Kelsey et al. 1986). Demographic variables such as age, education, and cultural background or ethnicity influence performance on neurobehavioral tests (Anger et al. 1997).

Years of education in one country may not be equivalent to years of education in another country (Puente 1990). A group’s familiarity with testing protocols or computers can also affect the validity of study findings.

There are several ways of handling these variables. During recruitment it is important to try to select groups that are comparable on age, education level, gender and ethnic background, and computers and to control for these variables in data analysis. Learning or practice effects may also confound interpretation of results. Studies that have assessed participants more than once have found improvements from the initial and subsequent testing that makes the determination of the effect of pesticide exposure alone very difficult (Bazylewicz-Walczak 1999; Daniell et al. 1992; Maizlish et al. 1987; Rohlman et al. 2001a). Strategies should be implemented to flatten the learning or practice effect before the exposure. Rohlman et al. (2001b) has done repeated testing of cognitive and motor performance with migrant farmworker children and found that there is significant practice effect between the first and second session but minimal change between the second and third session. Therefore, the use of the second pre-exposure measure would appear to be a valid baseline for comparison with performance postexposure.

### Across laboratory comparability

To build the case that specific functions are affected by pesticide exposure, it is important to have converging evidence. Although doubt may exist that it is possible to have reliable data that can be compared across studies, evidence suggests that such comparisons are possible. Anger et al. (1993) studied performance on neurobehavioral batteries conducted in 10 countries from four continents using the NCTB protocol. Although differences emerged, they could be explained by educational differences in the populations. However, performances on the six performance tests were remarkably similar across the nine countries in Europe, North America, and Asia that had similar educational levels.

Consistent findings of deficits on similar neuropsychological functions have been shown in studies of the same chemical exposure conducted by different investigators in different countries, languages, and cultures (Anger 1990, 2003). Most studies examining neurobehavioral performance and pesticide exposure have found that pesticide exposure is associated with deficits in cognitive and psychomotor function [see Kamel and Hoppin (2004) for a review]. However, an examination of the literature shows that some discrepancies do exist, different tests were affected in different studies, and in some cases no relationship between exposure and performance deficits was found.

These inconsistencies may be due to methodologic differences or to differences in exposure. Methodologic issues include the different formats and protocol used to assess neurobehavioral functioning in different populations. For example, 14 studies examining pesticide exposure included a variant of the digit span test (Table 2). Of these studies comparing exposed populations (defined by exposure or by occupational group), 4 showed significant deficits between exposed and control populations, 4 showed decrements in performance related to exposure, and 6 showed no decrements in performance. The digit span test demonstrates the difficulties when variants of the same test are used in different studies. The methodology used in this research needs to be standardized. Similar tests and protocols should be used to allow comparisons across studies.

Another important factor in explaining inconsistencies among studies is how exposure is defined (Alavanja et al. 2004). A range of exposure metrics, including living in an agricultural community (Cole et al. 1997), working on a farm (Fiedler et al. 1997; Kamel et al. 2003), specific job title (Bazylewicz-Walczak et al. 1999; Farahat et al. 2003; London et al. 1997; Roldan-Tapia et al. 2005), work history (Baldi et al. 2001), or acute exposure history (Rosenstock et al. 1991; Savage et al. 1988; Wesseling et al. 2002) have been used. The link between these classifications and actual exposure is often unknown, and it is possible that the amount of exposure in any given population varies considerably. To date, there have been no reports of an association between neuropsychological performance and any biological marker of exposure; however, organophosphate pesticides have a short half-life, and it is not likely that a short-term biomarker of exposure would be correlated with a cumulative effect on neurobehavioral performance. Biomarkers of effects of more cumulative exposures are needed.

Although there are many challenges in studying the farmworker population, using strict protocols, standardized measures, and quality-control procedures can help strengthen conclusions drawn from these studies and help to develop converging evidence. Equally important are the methods used for defining exposure. The variability in these methods can lead to incorrect conclusions. New and emerging biological techniques are being developed that will help identify exposed populations and allow accurate conclusions to be drawn.

## Needs and Emerging Techniques in the Measure of Health End Points

The ability to conduct large epidemiologic studies of health effects among the farmworker population is limited by access to health care and the migrant nature of the workforce. Markers of biological function offer opportunities to assess health effects among farmworker populations that are not dependent on health surveys or access to health records. Studies focusing on biologic tissues and mechanisms of action and incorporating gene–environment interactions are becoming increasingly more common.

The biomarker of a direct biological action resulting from exposure to organophosphate pesticides used most extensively with farmworker populations has been acetylcholinesterase (AChE) monitoring. Screening for cholinesterase inhibition as a result of exposure to organophosphate pesticides is mandated in California pesticide applicators and handlers; Washington State has recently implemented comparable legislation. Examining cholinesterase inhibition as a biomarker has advantages and disadvantages. Depression in cholinesterase activity can be observed at levels before clinical signs become apparent, leading to the early recognition of high-risk individuals and work operations (Wessels et al. 2003). But interpretation of AChE monitoring results is complicated by inter- and intraindividual variation in enzymatic activity and confounding factors. Exposure to large doses of organophosphate pesticides is required for significant AChE inhibition to occur, and therefore, it is more appropriately used as an indicator of toxicity at high exposure levels rather than low exposure levels (He 1999). In population-based studies, farmworkers have been found to have lower levels of AChE activity compared with individuals not employed in agriculture (Ciesielski 1994). No study, however, has reported a difference in family members of agricultural workers compared with controls even though urinary levels of pesticides and/or metabolites have been found to be higher in children of agricultural workers (Fenske et al. 2002; Loewenherz et al. 1997; Lu et al. 2001; Mills and Zahm 2001). Although AChE monitoring is invaluable in monitoring worker populations at high risk for acute pesticide exposure such as certified pesticide applicators and handlers, it not useful in monitoring health effects from low-dose chronic exposure to organophosphate pesticides among the large majority of farmworkers and their families.

New biological and genetic techniques are being developed that somewhat overcome the dependence on large study populations and longitudinal study designs and hold promise for studying health effects related to chronic low-level exposure to pesticides. These new techniques fall primarily into three areas: markers of DNA and RNA damage or repair, indicators of oxidative stress, and markers of changes in gene expression related to exposure to pesticides (Bolognesi 2003; Toraason et al. 2004). Many of these biomarkers are in a developmental status, have not been used extensively in agricultural populations, and lack evidence of an association between the biomarker and specific health outcomes. Nevertheless, they provide potential to increase our understanding of the biological mechanisms associated with the health outcomes that have been associated with pesticide exposures in multiple epidemiologic investigations.

### Markers of DNA damage

Exposure to pesticides has been associated with cancers, degenerative neurologic diseases, and altered immune response, but the mechanism of action is unclear. Genotoxic potential is a primary risk factor for long-term health effects such as cancer and reproductive health outcomes (Bolognesi 2003) Hagmar et al. (2001) reviewed the usefulness of cytogenetic biomarkers as intermediate end points in carcinogenesis and concluded that chromosomal aberration (CA) frequency predicts overall cancer risk in healthy subjects, but such associations have not been found for sister-chromatid exchanges and micronuclei (Mn). Although the genotoxic potential of pesticides is believed to be low, genotoxic monitoring in farmworker populations could be a useful tool to estimate genetic risk from exposure to complex pesticide mixtures over extended lengths of time. To date, genotoxic biomarker studies of workers exposed to pesticides have focused on cytogenetic end points, including CAs, Mn frequency, and sister-chromatid exchanges. In the last decade, single-cell gel electrophoresis or the comet assay has been established as a sensitive and rapid methods for the detection of DNA single-strand breaks and incomplete excision repair (Fairbain et al. 1995). These biomarkers have been well developed with high interlaboratory reliability, but they are not specific to pesticide exposure and to date have not been associated with a risk for human cancers or other disease outcomes.

In a review of the literature of pesticide exposure and DNA damage, Bolognesi (2003) reported a positive association between occupational exposure to complex pesticide mixtures and the presence of CAs, sister-chromatid exchanges, and Mn in most of the studies, but a number of studies failed to detect excess cytogenetic damage compared with control populations. The conflicting results from cytogenetic studies were attributed to the nature of the agricultural study populations and the type of exposure to pesticides. In general, data from one study in one particular occupational setting cannot be used to draw conclusions on genetic risk in another occupational setting. However, most studies on cytogenetic biomarkers in pesticide-exposed workers have indicated some dose-dependent effects, with increasing duration or intensity of exposure. The type of exposure can affect the results such as use versus nonuse of personal protective equipment and greenhouse workers versus open-field workers (Carbonell et al. 1993; Dulout et al. 1985; Falck et al. 1999). Studies have indicated that the persistence of chromosomal damage is short-lived for acute exposure (Eastmond 2000), and that damage may drop during low exposure periods for seasonal workers (Scarpato et al. 1996). However, multiple studies have indicated increased chromosomal damage associated with years of agricultural employment and year-round employment (Bolognesi et al. 1993; Gomez-Arroyo et al. 1992; Scarpato et al. 1996). Many of these new biomarkers will not provide a definitive answer linking exposure to disease; however, the use of these biomarkers could provide additional information to the weight of evidence that suggests a particular exposure is a potential health risk (Toraason et al. 2004). In some cases a specific genetic lesion that can be identified in an exposed population may be found, but this will not always be the case.

### Markers of cellular reaction to pesticides

The internal dose of a pesticide can be measured by concentration of the pesticide, its metabolites, or its reaction products. Reaction products (e.g., hemoglobin, albumin, and DNA adducts) can be viewed as an early biological effects or reactions that could lead to a potential health effect (Pirkle et al. 1995). Adducts can also be considered biomarkers of exposure (Costa 1996; Grissom 1995). These biomarkers reflect the dose of a certain agent or its metabolites that escapes detoxification and reaches its target protein or DNA. Adducts may form between blood components and toxicants such as pesticides when the toxicant reacts with the nucleophilic centers of nucleic acids (e.g., DNA) and proteins (e.g., hemoglobin and albumin) (Needham and Sexton 2000). These biochemical modifications precede structural or functional damage.

Oxidative damage is thought to be an important mechanism of damage for organophosphate pesticides (Banerjee et al. 2001; Halliwell 2002). Organophosphate pesticides can generate reactive oxygen species and alter cellular antioxidant systems (Bagchi et al. 1995; Delescluse et al. 2001; Flessel et al. 1993). Levels of products of oxidative damage in urine reflect overall damage to all tissues and organs in the body. More than 100 different oxidative modifications to DNA have been described (Loft and Poulsen 2001), and several DNA base oxidation products are known to be mutagenic, including 8-oxo-7,8-dihydro-2′-deoxyguanosine and thymine glycol (Halliwell 2002). The most studied and most abundant is the C-8 hydroxylation of the guanine base and glycol. Another potentially important mechanism for DNA damage, and ultimately cancer, is the generation of reactive species through peroxidation of lipids (Halliwell 2002; Marnett 2002). Malondialdehyde, one of the most abundant carbonyl products, can react with DNA to form adducts with deoxyguanosine, deoxyadenosine, and deoxycytidine that are mutagenic in bacterial and mammalian cells. As is the case with most of these biological markers of effect, specific oxidative modifications have not been associated with specific organophosphates and can be induced by multiple agents. Nonetheless, they offer significant potential in understanding the mechanism of action of these toxicologic agents and to make useful comparisons of exposure and potential health effects among exposure groups.

### Gene expression studies

The greatest potential for new biomarkers of early effect lies in toxicogenomics, a field of study that examines how the entire genome responds to toxicants or other hazards (Toraason et al. 2004). The ability to monitor changes in gene expression as a result of environmental exposure holds great promise in our understanding of the effect of environmental toxicants on human health. Ideally, gene expression studies will allow scientists to identify changes in transcription associated with exposure and subsequent risk of developing disease. Studies of the reaction of genes to an exposure usually results in thousands of genes showing altered expression patterns. Analysis of these changes in expression requires sophisticated data analysis techniques, storage, and mining strategies. These methods need to be developed before markers of changes in gene expression can be widely used in epidemiologic studies, but studies have been reported. For example, Infante-Rivard et al. (1999) conducted a case–control study of childhood leukemia and residential exposure to pesticides and examined gene–environment interactions, finding increased interaction odds ratios among carriers of the cytochrome P4501A1m1 (*CYP1A1m1*) and *CYP1a1m2* mutations when the mother during pregnancy or the child had been exposed to certain indoor insecticides. Longitudinal epidemiologic studies are needed to fully establish the predictive value of a change in gene expression and subsequent development of disease. Researchers who gain access to farmworker populations and who are able to follow them longitudinally should be encourage to bank genetic samples for future analyses as this area of science becomes more developed. These procedures would require that researchers be sensitive to the ethical, social, and legal issues related to obtaining genetic tissue for vulnerable, minority populations.

## Summary and Recommendations

Although there has been significant attention to the health effects of pesticides on human health, there has been little focus on the vulnerable farmworker population, and significant methodologic barriers make these studies extremely difficult. The leading obstacles are difficulties in establishing the population at risk and access to health information. The work environment contributes to the difficulty in ascertaining health status and their association with pesticide exposure. Improvements are needed in our ability to conduct surveillance of pesticide-related illnesses and worker compensation cases in this population. Language and education barriers contribute to this problem.

Neurobehavioral performance is the human health effect that has been most frequently identified after chronic organophosphate exposure. Although the measurement of neurobehavioral performance in non-English-speaking populations with limited education requires highly specialized techniques, the evidence to date points to a trend of decreased performance among farmworkers. Research on the risk of decreased performance among children of farmworkers is meager.

Emerging techniques in the development and use of biomarkers of health effects hold promise for improving our ability to study the effect of pesticide exposure in this population. These techniques include biomarkers of the biological action of the pesticide, markers of DNA and RNA damage or repair, and markers of changes in gene expression related to exposure to pesticides. Research is needed to improve our methods of exposure assessment and to establish the validity and reliability of these biological markers as predictors of subsequent health outcomes.

## Recommendations for Future Research

There is a critical need to link studies of exposure to pesticides to investigations of potential health effects. The barriers to studying health effects in this population have contributed to this lack of new knowledge regarding the health risks associated with pesticide exposure. Given the significant issues related to lack of national surveillance systems to capture the health status of farmworkers and disparities related to access to care, investigators should be encouraged to include biomarkers of health effects in their study designs. These studies are critical among occupational populations, but the children of farmworkers may be particularly vulnerable to the biological effects of pesticides.

Many studies of farmworkers suffer from small sample sizes. Researchers are encouraged to clearly define methods of assessing exposures and health effects in their research publications to allow comparisons to be made across studies and to conduct meta-analyses of similar studies.

Neurobehavioral testing remains an important measure to include in studies of farmworker populations. Large numbers of farmworkers and nonagricultural control groups need to be tested to provide normative values on the most common tests that are used in neurobehavioral testing. A meta-analysis of studies across geographic areas and among different exposure and age groups could provide significant evidence of the risk of neurobehavioral deficits among pesticide exposed populations.

Although maintaining study cohorts is a challenge, studies are needed that improve our ability to track this population over time. This is especially critical in assessing the impact of chronic low-dose exposure to pesticides and effects on neurobehavioral performance in younger populations. More birth cohorts of farmworker children need to be established. Strategies that have been used by investigators in Texas and California should be replicated.

In the future an increasing number of biomarkers will be available to assess both exposure and biological effects such as DNA damage, oxidative stress, and other biological mechanisms. These techniques coupled with measures of genetic susceptibility will improve our ability to characterize individual risk and to identify the more vulnerable members of the population. Effective communication will be needed to explain these tests to farmworker population and to provide appropriate risk communication. Given the challenges inherent in designing studies of the farmworker population, effective communication back to the farmworkers will increase future participation in research and optimally improve worker health.

## Figures and Tables

**Table 1:** Neurobehavioral effects studied in organophosphate-exposed populations.

Study	Population	Country
Wesseling et al. 2002	Banana farmers (poisoned)	Costa Rica
Cole et al. 1997	Farmers	Ecuador
Farahat et al. 2003	Cotton farmers	Egypt
Stephens et al. 1995	Sheep dippers	England
Stephens et al. 1996	Sheep dippers	England
Nishiwaki et al. 2001	Poisoning victims	Japan
Yokoyama et al. 1998	Poisoning victims	Japan
Rosenstock et al. 1991	Poisoning victims	Nicaragua
Miranda et al. 2002	Poisoning victims	Nicaragua
Bazylewicz-Walczak et al. 1999	Greenhouse workers	Poland
London et al. 1997	Fruit farmers	South Africa
Roldan-Tapia et al. 2005	Greenhouse workers	Spain
Kamel et al. 2003	Fern, nursery, fruit farmers	United States (Mexican immigrants)
Reidy et al. 1992	Farmers	United States (immigrant workers)
Rohlman et al. 2001b	Farmers	United States (Mexican immigrants)
Fiedler et al. 1997	Fruit farmers	United States
Savage et al. 1988	Poisoning victims	United States
Steenland et al. 1984	Poisoning victims	United States
Ruckart et al. 2004)	Overexposed children	United States
Korsak and Sato 1977	Farmers	United States
Maizlish et al. 1987	Pest control workers	United States

**Table 2:** Studies that have used variants of the digit span test to assess pesticide exposure.

Study	Method	Outcome
Bazylewicz-Walczak et al. 1999	Polish NCTB	−0
Cole et al. 1997	NCTB	~
Farahat et al. 2003	Unknown	+
Fiedler et al. 1997	WAIS-R	−0
Kamel et al. 2003	BARS	+
London et al. 1997	NCTB	−0
Nishiwaki et al. 2001	NCTB	~
Reidy et al. 1992	WAIS-R	~
Rohlman et al. 2001b	BARS	+
Rosenstock et al. 1991	WAIS-R	+
Stephens et al. 1995	Unknown	−0
Stephens et al. 1996	NES	−0
Wesseling et al. 2002	NCTB	~
Yokoyama et al. 1998	Japanese WAIS	−0

Abbreviations and symbols: +, poorer performance in exposed group; ~, nonsignificant trend observed with poorer performance in exposed group; 0, no significant difference between control and exposed groups; NES, Neurobehavioral Evaluation System; WAIS-R, Weschler Adult Intelligence Scale–Revised.

## References

[b1-ehp0114-000953] Alavanja MC, Hoppin JA, Kamel F (2004). Health effects of chronic pesticide exposure: cancer and neurotoxicity. Annu Rev Public Health.

[b2-ehp0114-000953] Anger WK (1990). Worksite behavioral research. Results, sensitive methods, test batteries and the transition from laboratory data to human health. Neurotoxicology.

[b3-ehp0114-000953] Anger WK 2003 Neurobehavioural tests and systems to assess neurotoxic exposures in the workplace and community Occup Environ Med 60 7 531 538 474. 12819291 10.1136/oem.60.7.531 PMC1740574

[b4-ehp0114-000953] Anger WK, Cassitto MG, Liang YX, Amador R, Hooisma J, Chrislip DW (1993). Comparison of performance from three continents on the WHO-recommended Neurobehavioral Core Test Battery. Environ Res.

[b5-ehp0114-000953] Anger WK, Letz R, Chrislip DW, Frumkin H, Hudnell K, Russo JM (1994). Neurobehavioral test methods for environmental health studies of adults. Neurotoxicol Teratol.

[b6-ehp0114-000953] Anger WK, Rohlman DS, Sizemore OJ, Kovera CA, Gibertini M, Ger J (1996). Human behavioral assessment in neurotoxicology: producing appropriate test performance with written and shaping instructions. Neurotoxicol Teratol.

[b7-ehp0114-000953] Anger WK, Sizemore OJ, Grossmann SJ, Glasser JA, Letz R, Bowler R (1997). Human neurobehavioral research methods: impact of subject variables. Environ Res.

[b8-ehp0114-000953] Arcury TA, Quandt SA, Mellen BG (2003). An exploratory analysis of occupational skin disease among Latino migrant and seasonal farmworkers in North Carolina. J Agric Saf Health.

[b9-ehp0114-000953] Bagchi D, Bagchi M, Hassoun EA, Stohs SJ (1995). *In vitro* and *in vivo* generation of reactive oxygen species, DNA damage and lactate dehydrogenase leakage by selected pesticides. Toxicology.

[b10-ehp0114-000953] Baldi I, Filleul L, Mohammed-Brahim B, Fabrigoule C, Dartigues JF, Schwall S (2001). Neuropsychologic effects of long-term exposure to pesticides: results from the French Phytoner study. Environ Health Perspect.

[b11-ehp0114-000953] Banerjee BD, Seth V, Ahmed RS (2001). Pesticide-induced oxidative stress: perspectives and trends. Rev Environ Health.

[b12-ehp0114-000953] Bazylewicz-Walczak B, Majczakowa W, Szymczak M (1999). Behavioral effects of occupational exposure to organophosphorous pesticides in female greenhouse planting workers. Neurotoxicology.

[b13-ehp0114-000953] Blair A, Hayes RB, Stewart PA, Zahm SH (1996). Occupational epidemiologic study design and application. Occup Med.

[b14-ehp0114-000953] Bolognesi C (2003). Genotoxicity of pesticides: a review of human biomonitoring studies. Mutat Res.

[b15-ehp0114-000953] Bolognesi C, Parrini M, Merlo F, Bonassi S (2003). Frequency of micronuclei in lymphocytes from a group of floriculturists exposed to pesticides. J Toxicol Environ Health.

[b16-ehp0114-000953] Calvert GM, Mehler LN, Rosales R, Baum L, Thomsen C, Male D (2003). Acute pesticide-related illnesses among working youths, 1988–1999. Am J Public Health.

[b17-ehp0114-000953] Carbonell E, Xamena N, Creus A, Marcos R (1993). Cytogenetic biomonitoring in a Spanish group of agricultural workers exposed to pesticides. Mutagenesis.

[b18-ehp0114-000953] Ciesielski S, Loomis DP, Mims SR, Auer A (1994). Pesticide exposures, cholinesterase depression, and symptoms among North Carolina migrant farmworkers. Am J Public Health.

[b19-ehp0114-000953] Cole DC, Carpio F, Julian J, Leon N, Carbotte R, De Almeida H (1997). Neurobehavioral outcomes among farm and nonfarm rural Ecuadorians. Neurotoxicol Teratol.

[b20-ehp0114-000953] Connan C 1996. Health Care Providers’ Knowledge of Pesticide Related Illness and Treatment. Seattle, WA:University of Washington.

[b21-ehp0114-000953] Cooper SP, Burau K, Hanis C, Henry J, MacNaughton N, Robison T (2001). Tracing migrant farmworkers in Starr County, Texas. Am J Ind Med.

[b22-ehp0114-000953] Cordes DH, Rea DF (1988). Health hazards of farming. Am Fam Physician.

[b23-ehp0114-000953] Costa LG (1996). Biomarker research in neurotoxicology: the role of mechanistic studies to bridge the gap between the laboratory and epidemiological investigations. Environ Health Perspect.

[b24-ehp0114-000953] Daniell W, Barnhart S, Demers P, Costa LG, Eaton DL, Miller M (1992). Neuropsychological performance among agricultural pesticide applicators. Environ Res.

[b25-ehp0114-000953] Daniels JL, Olshan AF, Savitz DA (1997). Pesticides and childhood cancers. Environ Health Perspect.

[b26-ehp0114-000953] Das R, Steege A, Baron S, Beckman J, Harrison R (2001). Pesticide-related illness among migrant farm workers in the United States. Int J Occup Environ Health.

[b27-ehp0114-000953] Delescluse C, Ledirac N, Li R, Piechocki MP, Hines RN, Gidrol X (2001). Induction of cytochrome P450 1A1 gene expression, oxidative stress, and genotoxicity by carbaryl and thiabendazole in transfected human HepG2 and lymphoblastoid cells. Biochem Pharmacol.

[b28-ehp0114-000953] Dulout FN, Pastori MC, Olivero OA, Gonzalez CM, Loria D, Matos E (1985). Sister-chromatid exchanges and chromosomal aberrations in a population exposed to pesticides. Mutat Res.

[b29-ehp0114-000953] Eastmond DA (2000). Benzene-induced genotoxicity: a different perspective. J Toxicol Environ Health.

[b30-ehp0114-000953] Ecobichon DJ 1996. Toxic effects of pesticides. In: Casarett and Doull’s Toxicology: The Basic Science of Poisons (Klaassen CD, Doull J, eds). 5th ed. New York:MacMillan, 643–689.

[b31-ehp0114-000953] Engel LS, O’Meara ES, Schwartz SM (2000). Maternal occupation in agriculture and risk of limb defects in Washington State, 1980–1993. Scand J Work Environ Health.

[b32-ehp0114-000953] Eskenazi B, Bradman A, Castorina R (1999). Exposures of children to organophosphate pesticides and their potential adverse health effects. Environ Health Perspect.

[b33-ehp0114-000953] Eskenazi B, Gladstone EA, Berkowitz GS, Drew CH, Faustman EM, Holland NT (2005). Methodologic and logistic issues in conducting longitudinal birth cohort studies: lessons learned from the Centers for Children’s Environmental Health and Disease Prevention Research. Environ Health Perspect.

[b34-ehp0114-000953] Fairbain DW, Olive PL, O’Neill KL (1995). The comet assay: a comprehensive review. Mutat Res.

[b35-ehp0114-000953] Falck GCM, Hirvonen A, Scarpato R, Saarikoski ST, Migliore L, Norppa H (1999). Micronuclei in blood lymphocytes and genetic polymorphism for GSTM1, GSTT1 and NAT2 in pesticide-exposed greenhouse workers. Mutat Res.

[b36-ehp0114-000953] Farahat TM, Abdelrasoul GM, Amr MM, Shebl MM, Farahat FM, Anger WK (2003). Neurobehavioural effects among workers occupationally exposed to organophosphorous pesticides. Occup Environ Med.

[b37-ehp0114-000953] Fenske RA, Lu C, Barr D, Needham L (2002). Children’s exposure to chlorpyrifos and parathion in an agricultural community in central Washington State. Environ Health Perspect.

[b38-ehp0114-000953] Firestone JA, Smith-Weller T, Franklin G, Swanson P, Longsteth WT, Checkoway H (2005). Pesticides and risk of Parkinson disease: a population-based case-control study. Arch Neurol.

[b39-ehp0114-000953] Fiedler N, Kipen H, Kelly-McNeil K, Fenske R (1997). Long-term use of organophosphates and neuropsychological performance. Am J Ind Med.

[b40-ehp0114-000953] Flessel P, Quintana PJE, Hooper K (1993). Genetic toxicity of malathion: a review. Enviorn Mol Mutagen.

[b41-ehp0114-000953] Garcia AM (2003). Pesticide exposure and women’s health. Am J Ind Med.

[b42-ehp0114-000953] Gomez-Arroyo S, Noriega-Aldana N, Osorio A, Galicia F, Ling S, Villalobos-Pietrini R (1992). Sister-chromatid exchange analysis in a rural population of Mexico exposed to pesticide. Mutat Res.

[b43-ehp0114-000953] Graber DR, Musham C, Bellack JP, Holmes D (1995). Environmental health in medical school curricula: views of academic deans. J Occup Environ Med.

[b44-ehp0114-000953] Hagmar L, Stromberg U, Tinnerberg H, Mikoczy Z (2001). The usefulness of cytogenetic biomarkers as intermediate endpoints in carcinogenesis. Int J Hygiene Environ Health.

[b45-ehp0114-000953] Halliwell B (2002). Effect of diet on cancer development: is oxidative DNA damage a biomarker?. Free Radic Biol Med.

[b46-ehp0114-000953] Hanke W, Jurewicz J (2004). The risk of adverse reproductive and developmental disorders due to occupational pesticide exposure: an overview of current epidemiological evidence. Int J Occup Med Environ Health.

[b47-ehp0114-000953] Hansen E, Donohoe M (2003). Health issues of migrant and seasonal farmworkers. J Health Care Poor Underserved.

[b48-ehp0114-000953] He F (1999). Biological monitoring of exposure to pesticides: current issues. Toxicol Lett.

[b49-ehp0114-000953] Infante-Rivard C, Labuda D, Krajinovic M, Sinnett D (1999). Risk of childhood leukemia associated with exposure to pesticides and with gene polymorphisms. Epidemiology.

[b50-ehp0114-000953] Iregren A, Gamberale F, Kjellberg A (1996). SPES: a psychological test system to diagnose environmental hazards. Swedish Performance Evaluation System. Neurotoxicol Teratol.

[b51-ehp0114-000953] Jaga K, Dharmani C (2003). Sources of exposure to and public health implications of organophosphate pesticides. Pan Am J Public Health.

[b52-ehp0114-000953] Johnson BL Baker EL El Batawi M Gilioli R Hanninen H Seppalainen AM et al., eds. 1987. Prevention of Neurotoxic Illness in Working Populations. New York:John Wiley & Sons.

[b53-ehp0114-000953] Kamel F, Hoppin JA (2004). Association of pesticide exposure with neurologic dysfunction and disease. Environ Health Perspect.

[b54-ehp0114-000953] Kamel F, Rowland AS, Park LP, Anger WK, Baird DD, Gladen BC (2003). Neurobehavioral performance and work experience in Florida farmworkers. Environ Health Perspect.

[b55-ehp0114-000953] Kelsey JL Thompson WD Evans AS 1986. Methods in Observational Epidemiology. New York:Oxford University Press.

[b56-ehp0114-000953] Korsak RJ, Sato MM (1977). Effects of organophosphate pesticides chronic exposure on the central nervous system. Clin Toxicol.

[b57-ehp0114-000953] Letz R 1990. The Neurobehavioral Evaluation System: an international effort. In: Advances in Neurobehavioral Toxicology: Applications in Environmental and Occupational Health (Johnson BL, ed). Chelsea, MI:Lewis Publishers, 189–201.

[b58-ehp0114-000953] Loewenherz C, Fenske RA, Simcox NJ, Bellamy G, Kalman D (1997). Biological monitoring of organophosphorus pesticide exposure among children of agricultural workers in central Washington State. Environ Health Perspect.

[b59-ehp0114-000953] Loft S, Poulsen HE (2000). Antioxidant intervention studies related to DNA damage, DNA repair and gene expression. Free Radic Res.

[b60-ehp0114-000953] London L, Myers JE, Nell V, Taylor T, Thompson ML (1997). An investigation into neurologic and neurobehavioral effects of long-term agrichemical use among deciduous fruit farm workers in the Western Cape, South Africa. Environ Res.

[b61-ehp0114-000953] Lu C, Knutson DE, Fisker-Andersen J, Fenske RA (2001). Biological monitoring survey of organophosphorus pesticide exposure among pre-school children in the Seattle metropolitan area. Environ Health Perspect.

[b62-ehp0114-000953] Maizlish N, Schenker M, Weisskopf C, Seiber J, Samuels S (1987). A behavioral evaluation of pest control workers with short-term, low-level exposure to the organophosphate diazinon. Am J Ind Med.

[b63-ehp0114-000953] Marnett LJ (2002). Oxy radicals, lipid peroxidation and DNA damage. Toxicology.

[b64-ehp0114-000953] McCauley LA, Lasarev MR, Higgins G, Rothlein J, Muniz J, Ebbert C (2001). Work characteristics and pesticide exposures among migrant agricultural families: a community-based research approach. Environ Health Perspect.

[b65-ehp0114-000953] McCauley LA, Sticker D, Bryan C, Lasarev MR, Scherer JA (2002). Pesticide knowledge and risk perception among adolescent Latino farmworkers. J Agric Saf Health.

[b66-ehp0114-000953] McCurdy SA, Samuels SJ, Carroll DJ, Beaumont JJ, Morrin LA (2002). Injury risks in children of California migrant Hispanic farm worker families. Am J Ind Med.

[b67-ehp0114-000953] Mc Donald M 2001. Recruitment, retention and training of bilingual/bicultural staff. In: Migrant Health Issues Monograph Series (National Advisory Council on Migrant Health, ed). Buda, TX:National Center For Farmworker Health Inc., 45–50.

[b68-ehp0114-000953] Mills PK, Zahm SH (2001). Organophosphate pesticide residues in urine of farmworkers and their children in Fresno County, California. Am J Ind Med.

[b69-ehp0114-000953] Miranda J, McConnell R, Delgado E, Cuadra R, Keifer M, Wesseling C (2002). Tactile vibration thresholds after acute poisonings with organophosphate insecticides. Int J Occup Environ Health.

[b70-ehp0114-000953] Moses M (1989). Pesticide-related health problems and farmworkers. AAOHN.

[b71-ehp0114-000953] National Center for Farmworker Health. 2004. Facts about Farmworkers. Available: http://www.ncfh.org/docs/fs-Facts%20about%20Farmworkers.pdf [accessed 30 September 2004].

[b72-ehp0114-000953] National Environmental Education and Training Foundation. 2002. National Strategies for Health Care Providers: Pesticides Initiative. Implementation Plan. Washington, DC:National Environmental Education and Training Foundation. Available: http://www.neetf.org/Health/providers/implplan.shtm [accessed 14 December 2004].

[b73-ehp0114-000953] Needham LL, Sexton K (2000). Assessing children’s exposure to hazardous environmental chemicals: an overview of selected research challenges and complexities. J Expo Anal Environ Epidemiol.

[b74-ehp0114-000953] Nishiwaki Y, Maekawa K, Ogawa Y, Asukai N, Minami M, Omae K, The Sarin Health Effects Study Group (2001). Effects of sarin on the nervous system in rescue team staff members and police officers 3 years after the Tokyo subway sarin attack. Environ Health Perspect.

[b75-ehp0114-000953] Nordstrom DL, Krauska M, DeStefano F, Colt JS, Zahm SH (2001). Ability to trace migrant farmworkers ten years after initial identification in a northern state (Wisconsin). Am J Ind Med.

[b76-ehp0114-000953] Olsen GW, Brondum J, Bodner KM, Kravat BA, Mandel JS, Mandel JH (1990). Occupation and industry on death certificates of long-term chemical workers: concordance with work history records. Am J Ind Med.

[b77-ehp0114-000953] O’Malley MA (1997). Skin reactions to pesticides. Occup Med.

[b78-ehp0114-000953] Pirkle JL, Needham LL, Sexton K (1995). Improving exposure assessment by monitoring human tissues for toxic chemicals as part of a National Human Exposure Assessment Survey. J Expo Anal Environ Epidemiol.

[b79-ehp0114-000953] Priyadarsi A, Khuder SA, Schaub EA, Shrivastava S (2000). A meta-analysis of Parkinsons disease and expsoure to pesticides. Neurotoxicology.

[b80-ehp0114-000953] Puente AE Mora MS Munoz-Cespedes JM 1990. Neuropsychological assessment of Spanish-speaking children and youth. In: Handbook of Clinical Child Neuropsychology (Reynolds CR, Fletcher E, eds). New York:Plenum Press, 371–383.

[b81-ehp0114-000953] Quandt SA, Preisser JS, Arcury TA (2002). Mobility patterns of migrant farmworkers in North Carolina: implications for occupational health research and policy. Hum Organ.

[b82-ehp0114-000953] Reidy TJ, Bowler RM, Rauch SS, Pedroza GI (1992). Pesticide exposure and neuropsychological impairment in migrant farm workers. Arch Clin Neuropsychol.

[b83-ehp0114-000953] Rohlman DS, Anger WK, Tamulinas A, Phillips J, Bailey SR, McCauley L (2001a). Development of a neurobehavioral battery for children exposed to neurotoxic chemicals. Neurotoxicology.

[b84-ehp0114-000953] Rohlman DS, Bailey SR, Anger WK, McCauley L (2001b). Assessment of neurobehavioral function with computerized tests in a population of Hispanic adolescents working in agriculture. Environ Res.

[b85-ehp0114-000953] Rohlman DS, Gimenes LS, Eckerman DA, Kang SK, Farahat FM, Anger WK (2003). Development of the Behavioral Assessment and Research System (BARS) to detect and characterize neurotoxicity in humans. Neurotoxicology.

[b86-ehp0114-000953] Roldan-Tapia L, Parron T, Sanchez-Santed F (2005). Neuropsychological effects of long-term exposure to organophosphate pesticides. Neurotoxicol Teratol.

[b87-ehp0114-000953] Rosenstock L, Keifer M, Daniell WE, McConnell R, Claypoole K (1991). Chronic central nervous system effects of acute organophosphate pesticide intoxication. The Pesticide Health Effects Study Group. Lancet.

[b88-ehp0114-000953] Ruckart PZ, Kakolewski K, Bove FJ, Kaye WE (2004). Long-term neurobehavioral health effects of methyl parathion exposure in children in Mississippi and Ohio. Environ Health Perspect.

[b89-ehp0114-000953] Savage EP, Keefe TJ, Mounce LM, Heaton RK, Lewis JA, Burcar PJ (1988). Chronic neurological sequelae of acute organophosphate pesticide poisoning. Arch Environ Health.

[b90-ehp0114-000953] Scarpato R, Migliore L, Angotzi G, Fedi A, Miligi L, Loprieno N (1996). Cytogenetic monitoring of a group of Italian floriculturists. No evidence of DNA damage related to pesticide exposure. Mutat Res.

[b91-ehp0114-000953] Schenk M, Popp SM, Neale AV, Demers RY (1996). Environmental medicine content in medical school curricula. Acad Med.

[b92-ehp0114-000953] Schwartz DA, Newsum LA, Heifetz RM (1986). Parental occupational and birth outcome in an agricultural community. Scand J Work Environ Health.

[b93-ehp0114-000953] Seid M, Castaneda D, Mize R, Zivkovic M, Varni JW (2003). Crossing the border for health care: access and primary care characteristics for young children of Latino farm workers along the US-Mexico border. Ambul Pediatr.

[b94-ehp0114-000953] Stallones L, Beseler C (2002). Pesticide illness, farm practices, and neurological symptoms among farm residents in Colorado. Environ Res.

[b95-ehp0114-000953] Steenland K, Beaumont J (1984). The accuracy of occupation and industry data on death certificates. J Occup Med.

[b96-ehp0114-000953] Stephens R, Spurgeon A, Berry H (1996). Organophosphates: the relationship between chronic and acute exposure effects. Neurotoxicol Teratol.

[b97-ehp0114-000953] Stephens R, Spurgeon A, Calvert IA, Beach J, Levy LS, Berry H (1995). Neuropsychological effects of long-term exposure to organophosphates in sheep dip. Lancet.

[b98-ehp0114-000953] Strong LL, Thompson B, Coronado GD, Griffith WC, Vigoren EM, Islas I (2004). Health symptoms and exposure to organophosphate pesticides in farmworkers. Am J Ind Med.

[b99-ehp0114-000953] Thompson B, Coronado GD, Grossman JE, Puschel K, Solomon CC, Islas I (2003). Pesticide take-home pathway among children of agricultural workers: study design, methods, and baseline findings. J Occup Environ Med.

[b100-ehp0114-000953] Toraason M, Albertini R, Bayard S, Bigbee W, Blair A, Boffetta P (2004). Applying new biotechnologies to the study of occupational cancer—a workshop summary. Environ Health Perspect.

[b101-ehp0114-000953] Van Maele-Fabry G, Willems JL (2003). Occupation related pesticide exposure and cancer of the prostate: a meta-analysis. Occup Environ Med.

[b102-ehp0114-000953] Villarejo D (2003). The health of U.S. hired farm workers. Annu Rev Public Health.

[b103-ehp0114-000953] Villarejo D Lighthall D Williams D Bade B Samuels S Mc Curdy S 2000. Suffering in Silence: A Report on the Health of California’s Agricultural Workers. Davis, CA:California Endowment.

[b104-ehp0114-000953] Wesseling C, Keifer M, Ahlbom A, McConnell R, Moon JD, Rosenstock L (2002). Long-term neurobehavioral effects of mild poisonings with organophosphate and n-methyl carbamate pesticides among banana workers. Int J Occup Environ Health.

[b105-ehp0114-000953] Wessels D, Barr DB, Mendola P (2003). Use of biomarkers to indicate exposure of children to organophosphate pesticides: implications for a longitudinal study of children’s environmental health. Environ Health Perspect.

[b106-ehp0114-000953] Yokoyama K, Araki S, Murata K, Nishikitani M, Okumura T, Ishimatsu S (1998). Chronic neurobehavioral effects of Tokyo subway sarin poisoning in relation to posttraumatic stress disorder. Arch Environ Health.

[b107-ehp0114-000953] Zahm SH, Blair A (1993). Cancer among migrant and seasonal farmworkers: an epidemiologic review and research agenda. Am J Ind Med.

[b108-ehp0114-000953] Zahm SH, Blair A (2001). Assessing the feasibility of epidemiologic research on migrant and seasonal farmworkers: an overview. Am J Ind Med.

